# Globally applicable solution to hearing loss screening: a diagnostic accuracy study of tablet-based audiometry

**DOI:** 10.1136/bmjopen-2024-097550

**Published:** 2025-05-22

**Authors:** Jamie Cheong, Emily Lowe, Chang Woo Lee, Claudia Barbosa, Lise Gillen, Emma King, Presanna Premachandra, Anand Shah, Francis Drobniewski

**Affiliations:** 1 Imperial College London, London, UK; 2 Royal Brompton and Harefield Hospitals, London, UK; 3 University Hospitals Dorset NHS Foundation Trust, Poole, UK; 4 Guy's and St Thomas’ NHS Foundation Trust, London, UK

**Keywords:** Health Equity, Hearing, Audiology, Pharmacists, PUBLIC HEALTH, Toxicity

## Abstract

**Objectives:**

Hearing loss (HL) affects 20% of the world’s population, with shortages of audiologists and audiometric sound booths unable to meet demand for hearing care services. We aimed to assess the accuracy of tablet-based audiometry (TA) to screen for HL at standard (0.25–8 kHz) and extended high frequencies (>8 kHz).

**Design:**

Diagnostic accuracy study.

**Setting:**

Two secondary care audiology and ear, nose and throat outpatient clinics in the UK between April 2022 and September 2023.

**Participants:**

Adults aged≥16 years undergoing sound booth audiometry (SBA).

**Interventions:**

TA, hearing-related questionnaires and patient usability questionnaires.

**Outcome measures:**

Sensitivity, specificity and accuracy of TA compared with SBA for detecting HL. Patient usability assessment of TA and SBA.

**Results:**

129 patients were enrolled with 127 patients (254 ears) included in the final analysis. Median age was 43 years (IQR 33–56), 55% (70/127) were women. 76% (96/127) and 68% (86/127) of patients had HL defined by British Society of Audiology (BSA) and American Speech–Language–Hearing Association (ASHA) criteria. Age was significantly associated with HL (p<0.0001); however, hearing-related questionnaire scores were not significantly different between those with or without HL. There was no significant difference in detecting HL between TA and SBA using either BSA or ASHA criteria at each frequency. Overall, 92% (1612/1751) of TA results were within 10 dB agreement with SBA results. Sensitivity and specificity of TA for detecting HL were 77–100% and >85%, respectively, between 0.25 and 12.5 kHz. In terms of patient usability, TA showed significantly higher scores in attractiveness (p<0.0001), novelty (p<0.0001), efficiency (p=0.0003), stimulation (p=0.003) and perspicuity (p=0.02).

**Conclusions:**

TA demonstrated good sensitivity with high specificity for detecting HL at frequencies 0.25–12.5 kHz and would be an acceptable accurate alternative to SBA. This would increase the accessibility of HL screening and has the potential to be used as a diagnostic test in those without tinnitus where resources are limited.

**Trial registration number:**

NCT05847556.

**STRENGTHS AND LIMITATIONS OF THIS STUDY**

- Prospective multicentre study measuring accuracy of tablet-based audiometry at both standard frequencies (0.25–8 kHz) and extended high frequencies (EHF, >8 kHz).
- Patient usability of a novel device is reported.
- Fewer paired test results at EHF were due to a lack of facilities measuring this frequency range.
- Ambient environmental noise was not measured.

## Introduction

 Hearing loss (HL) is a reduced perception of sound, defined by the WHO as hearing thresholds≥20 dB, which in 2019, affected 1.57 billion people or, 20.3% of the global population.^1 2^ The incidence of HL increases with advancing age, with 42% of those over 60 years having hearing impairment caused by natural degenerative changes in the ear, lifetime ototoxic injuries, genetic susceptibility and/or modifiable lifestyle behaviours. More than 50% of HL is preventable (ear infections, vaccine-preventable illness, exposure to noise, chemicals and medications).^1^ Intravenous aminoglycoside (AG) antibiotics remain a cornerstone of infection treatment and are used globally, especially in patients with chronic respiratory infections such as those with multidrug-resistant tuberculosis (MDRTB) and other mycobacterial infections.^3^ Irreversible ototoxicity is a known side effect of intravenous AG; the global prevalence of HL associated with exposure to short courses of intravenous AG (<16 days) is 16.6% and for MDRTB is 40.6%.^4^ Despite their widespread use, systems for identifying early HL in low-income or middle-income countries (LMICs) are scarce.^5^

Delayed diagnosis of HL in children can cause speech and language development issues continuing into adulthood, with children less likely to go onto higher education, more likely to be unemployed, have poorer mental health, lower quality of life and social isolation affecting cognition. HL is the second highest modifiable risk factor (after depression) for enhancing dementia-related problems.^1 6^ HL is associated with increased healthcare expenditure, loss of productivity and reduced quality of life with an estimated global cost exceeding $981 billion in 2019.^7^

The gold standard in high-income countries (HICs) for monitoring hearing is testing by audiologists within audiometric sound booths, which reduce ambient noise and assess HL typically within speech range frequencies (0.25–8 kHz).^8^ However, the increased demand on audiology services, along with a shortage of audiologists, has led to HICs being unable to meet existing demand.^9 10^ LMICs in particular have a scarcity of audiologists (78% of countries in Africa have less than one audiologist per one million population) compared with HICs (52% of countries in Europe have ten audiologists per one million population).^1^ The expense of installing audiometric booths also limits availability.^11^ Age-related, noise-induced and drug-induced HL initially occurs at extended high frequencies (EHF, >8 kHz) before affecting speech range frequencies, and hence, EHF monitoring is recommended for early detection in high-risk populations, allowing for alternative drug treatment regimens, reductions in noise exposure and aural rehabilitation.^12^,^16^ A 5% reduction in the prevalence of HL has been estimated to reduce global costs by US$49 billion.^7^ However, at present, EHFs are not routinely monitored during standard sound booth audiometry (SBA) with HL detection occurring only after progression to speech range frequencies.

Boothless audiometry using mobile technology could provide a solution to overcome the challenges of availability, cost and accessibility to the limited numbers of sound booths in LMICs and HICs.^1 17^ Tablet-based audiometry (TA) using automated technology also reduces operator training requirements and allows trained staff other than audiologists to provide surveillance screening services.^17^ Circumaural transducers used without booths for monitoring EHFs have good noise attenuation and could improve accessibility to hearing screening and achieve earlier diagnosis of HL.^18^ Boothless audiometry measuring EHF is therefore required to detect HL in ototoxic drug and noise exposure or where SBA is unavailable. TA using Shoebox has been validated in other studies in children and adults attending audiology outpatients, emergency departments, or patients with cognitive impairment, at frequencies up to 8 kHz.^19^,^26^ Our previous work has demonstrated the use of TA as an accurate screening tool in individuals with cystic fibrosis (CF) up to frequencies of 12.5 kHz.^27^ Unlike most other published studies, we have conducted a multicentre study, analysing TA accuracy compared with SBA at both standard frequencies (0.25–8 kHz) and EHF up to 16 kHz, in a general audiology and ear, nose and throat (ENT) outpatient clinic setting. Additionally, we have also focused on assessing the TA and SBA approaches from the patients’ perspective, including the usability of both audiometric processes.

## Methods

### Study design and procedures

Audiometry and data collection were performed prospectively in this diagnostic accuracy study conforming to STARD (Standards for Reporting Diagnostic Accuracy) guidelines of the Shoebox Standard Limited portable audiometer compared with SBA as the gold standard.^28^ Patients were recruited from audiology and ENT outpatient clinics at Guy’s & St Thomas’ NHS Foundation Trust and University Hospitals Dorset NHS Foundation Trust across two locations in the UK. The study was registered on clinicaltrials.gov (NCT05847556), where the full protocol can be accessed.

Demographic information, medical and drug history, referral, reason for audiometry if known were recorded. Data were entered into a RedCap database (V.14.1.4). Definition of HL was defined by the British Society of Audiology (BSA) as thresholds>20 dB and American Speech–Language–Hearing Association (ASHA) standards as thresholds>25 dB.^15 29^ Scores for hearing-related questionnaires were assigned: no (0 points), sometimes (2 points) and yes (4 points) with total scores categorised into different severities.^30^,^32^

Any patient aged 16 years or above attending the ENT and audiology clinics, between April 2022 and September 2023, who consented was eligible to participate. Those exposed to ototoxic medicines or noise and who agreed to take part in the study were also included and listed in the online supplemental material. Those who were aged<16 years or unable to provide informed consent were excluded. SBA was carried out by an audiologist in a sound-attenuated booth/room. TA was self-administered by the patient with supervision by another staff member not carrying out SBA (pharmacist, ENT doctor, audiologist) in a quiet clinic room. Objective measures do not always correlate with subjective symptoms, and disability experienced varies among individuals with the same disease.^33^ Use of questionnaires to assess disease burden can help address symptoms that are not identified by standard hearing tests. We wanted to observe if the presence of tinnitus affects TA performance and how TA compares to hearing-related questionnaires for HL screening. Validated hearing-related questionnaires were completed by patients in between SBA and TA sessions: Hearing Handicap Inventory for Adults (HHIA, 25-item assessment of hearing impairment on emotional, social adjustments in adults), and if experiencing symptoms of tinnitus or dizziness, Tinnitus Handicap Inventory (THI, 25-item reviewing functional, emotional and catastrophic disability scales) and Dizziness Handicap Inventory (DHI, 25-item evaluating functional, emotional and physical domains).^34^,^36^ They were also asked to fill in the User Experience Questionnaire (UEQ) about their experience of TA and SBA, which is a quick validated tool to measure the user’s experience.^37^ The UEQ assesses hedonic and pragmatic quality aspects involving 26 polarised statements graded on a 7-point Likert scale to gauge patient opinions on six scales: attractiveness, perspicuity, dependability, efficiency, novelty and stimulation. This was created on a Qualtrics survey to produce an electronic form. Patients completed two UEQs (one for each type of audiometry) after finishing both audiometry sessions. As SBA was part of the patient’s standard of care, 30–45 min was allowed for TA, ototoxicity questionnaires and UEQs. Audiologists carrying out SBA were blinded to the TA results.

The clinical audiometer used in SBA was the Natus Aurical calibrated according to BSA standards.^29^ TA was carried out using Shoebox Standard edition software application on Apple iPads with circumaural Radioear DD450 transducers measuring frequencies 0.25–16 kHz, calibrated by Shoebox Limited to comply with American National Standards Institute standards (S3.6-1996-2010). Adult pure tone automated test mode was selected on the Shoebox Standard application, which uses a Modified Hughson Westlake algorithm. Hearing thresholds for 0.25, 0.5, 1, 2, 4, 6, 8, 10, 12.5 and 16 kHz were compared between TA and SBA.

### Statistical analysis

Sample size calculation was performed to measure sensitivity and specificity of TA compared with SBA with an expected sensitivity at 90% and specificity of 85%, with 95% CI, using an estimated prevalence of HL higher than the normal population of 50% given the cohort being referred for ENT review and an expected 10% dropout rate. This revealed that a minimum of 109 patients was required to achieve statistical power. Statistical analyses were performed using GraphPad Prism V.10.1.1 (270). Right and left ear thresholds were combined for each frequency. Mean/median was calculated for parametric/non-parametric data. Χ^2^ or Fisher’s exact tests were used for categorical data. TA measurements were compared with SBA results using Bland-Altman plots to visually assess agreement, correlation and paired t-tests to observe the differences between the two types of measurements, and simple linear regression was used to determine the presence of proportional bias. Cronbach’s alpha test was used to assess reliability. Sensitivity, specificity, positive and negative predictive values (PPV and NPV) with 95% CIs were calculated. Statistical significance was defined as p<0.05. Usability was analysed using www.ueq-online.org, where data analysis tools for UEQ are available.

### Patient and public involvement

Patients and the public were not involved in the design, conduct, reporting or dissemination plans of this research.

## Results

Between 16 March 2022 and 15 September 2023, 129 patients were enrolled with 127 patients (254 ears) included in the final analysis (2 patients were excluded—1 was pregnant, the SBA and TA were more than 1 week apart for another patient) (online supplemental figure S1). The number of possible tests and results available for data analysis are shown in online supplemental table S1. In total, 98% (124/127) of patients carried out TA on the same day as SBA, with 2% (3/127) of TA performed within 5 days of SBA.

Table 1 shows the demographic data and the incidence of HL based on BSA (76%) and ASHA (68%) thresholds: median age was 43 years (IQR 33–56), 70 (55%) were women, 79 (62%) were white British. 91 patients (72%) were referred by ENT and primary reasons for SBA were related to middle ear symptoms (24%) or dizziness/vertigo/balance issues (17%) (online supplemental tables S2 and S3). Most common concurrent medication with ear-related side effects were antidepressants (12%) and 42% of patients had received aspirin or NSAIDs in the previous 3 months (online supplemental table S4). Age was significantly associated with HL regardless of the criteria used; all other characteristics were not (sex, ethnicity, hearing-related questionnaire scores) with no significant difference in HL between BSA and ASHA thresholds (table 1). Based on BSA criteria in patients without apparent HL, 39% (12/31), 39% (12/31) and 58% (18/31) had HL, tinnitus and dizziness symptoms, respectively, causing at least mild handicap or above based on questionnaire scores of HHIA>16, THI>16 and DHI>0. A similar trend was observed with ASHA criteria at 41% (17/41), 39% (16/41) and 51% (21/41), respectively, in patients with no HL.

**Table 1:** Demographic patient data for frequencies 0.25–8 kHz

Characteristic	N (%) or median (IQR)	Individuals with HL as per BSA threshold (%)	No HL (%)	P value	Individuals with HL as per ASHA threshold (%)	No HL(%)	P value
Patients (n)	127 (100)	96 (76)	31 (24)		86 (68)	41 (32)	0.21
Median age of patients, years	43 (33–56)	47 (36–60)	35 (26–43)	**<0.0001**	48 (37–60)	35 (27–43)	**<0.0001**
Female	70 (55)	54 (56)	16 (52)	0.68	48 (56)	22 (54)	0.85
HHIA				0.10			0.09
0–16 (no handicap)	58 (46)	39 (41)	19 (61)		34 (40)	24 (59)	
18–42 (mild–moderate handicap)	43 (34)	37 (39)	6 (19)		34 (40)	9 (22)	
44–100 (significant handicap)	26 (20)	20 (21)	6 (19)		18 (21)	8 (20)	
THI				0.51			0.23
No symptoms	38 (30)	27 (28)	11 (35)		23 (27)	15 (37)	
0–16 (slight/no handicap)	36 (28)	28 (29)	8 (26)		26 (30)	10 (24)	
18–36 (mild handicap)	16 (13)	11 (11)	5 (16)		10 (12)	6 (15)	
38–56 (moderate handicap)	17 (13)	12 (13)	5 (16)		10 (12)	7 (17)	
58–76 (severe handicap)	11 (9)	9 (9)	2 (6)		8 (9)	3 (7)	
78–100 (catastrophic handicap)	0 (0)	0 (0)	0 (0)		0 (0)	0 (0)	
Missing data	9 (7)	9 (9)	0 (0)		9 (10)	0 (0)	
DHI				0.23			0.66
No symptoms	69 (54)	57 (59)	12 (39)		50 (58)	19 (46)	
0–30 (mild)	35 (28)	24 (25)	11 (35)		22 (26)	13 (32)	
31–60 (moderate)	15 (12)	9 (9)	6 (19)		9 (10)	6 (15)	
61–100 (severe)	4 (3)	3 (3)	1 (3)		2 (2)	2 (5)	
Missing data	4 (3)	3 (3)	1 (3)		3 (3)	1 (2)	
Ethnicity (patients)				0.23			0.26
African	5 (4)	2 (2)	3 (10)		2 (2)	3 (7)	
Bangladeshi	1 (1)	1 (1)	0 (0)		1 (1)	0 (0)	
Black other	1 (1)	1 (1)	0 (0)		1 (1)	0 (0)	
Caribbean	5 (4)	4 (4)	1 (3)		3 (3)	2 (5)	
Chinese	2 (2)	1 (1)	1 (3)		1 (1)	1 (2)	
Indian	4 (3)	2 (2)	2 (6)		1 (1)	3 (7)	
Other	4 (3)	2 (2)	2 (6)		1 (1)	3 (7)	
Other mixed background	4 (3)	2 (2)	2 (6)		2 (2)	2 (5)	
Unspecified	1 (1)	1 (1)	0 (0)		1 (1)	0 (0)	
White and Asian	1 (1)	0 (0)	1 (3)		0 (0)	1 (2)	
White and Black African	2 (2)	1 (1)	1 (3)		1 (1)	1 (2)	
White and Black Caribbean	2 (2)	1 (1)	1 (3)		1 (1)	1 (2)	
White British	79 (62)	66 (69)	13 (42)		60 (70)	19 (46)	
White Irish	2 (2)	2 (2)	0 (0)		2 (2)	0 (0)	
White other	14 (11)	10 (10)	4 (13)		9 (10)	5 (12)	

Bold p values are statistically significant

ASHA, American Speech-Language-Hearing Association>25 dB; BSA, British Society of Audiology>20 dB; DHI, Dizziness Handicap Inventory; HHIA, Hearing Handicap Inventory for Adults; HL, hearing loss; THI, Tinnitus Handicap Inventory.

There was no significant difference in detecting HL between TA and SBA using either BSA or ASHA criteria (table 2). Mean pure tone thresholds for each frequency for TA and SBA are shown in table 3. Subanalysis of mean pure tone paired thresholds (online supplemental tables S5 and S6) comparing only where SBA thresholds were abnormal according to BSA (>20 dB) and ASHA (>25 dB) criteria for HL revealed that six (0.5, 1, 2, 6, 8 and 10 kHz) and five (0.5, 2, 6, 8 and 10 kHz) thresholds, respectively, remain significantly different compared with seven thresholds (0.25–4, 8 and 12.5 kHz) of all paired thresholds (table 3). TA results were highly correlated for most frequencies (0.25–12.5 kHz) but not directly comparable to SBA results (except at 6, 10 and 16 kHz) (table 3). 92% of TA results, however, were within 10 dB agreement with SBA results, highlighting agreement for most frequencies (online supplemental table S7).

**Table 2:** Hearing loss detected at pure tone thresholds according to BSA and ASHA criteria

Frequency (kHz)	Paired results	BSA (>20 dB)			ASHA (>25 dB)		
		TA, n, (%)	SBA, n, (%)	P value	TA, n, (%)	SBA, n, (%)	P value
0.25	231	54 (23)	56 (24)	0.91	40 (17)	39 (17)	1.00
0.5	231	53 (23)	62 (27)	0.39	41 (18)	46 (20)	0.63
1	232	56 (24)	63 (27)	0.52	40 (17)	42 (18)	0.90
2	233	61 (26)	70 (30)	0.41	44 (19)	45 (19)	1.00
4	230	107 (47)	104 (45)	0.85	85 (37)	84 (37)	1.00
6	228	95 (42)	111 (49)	0.16	84 (37)	91 (40)	0.56
8	222	103 (46)	94 (42)	0.44	91 (41)	83 (37)	0.50
10	72	36 (50)	39 (54)	0.74	30 (42)	37 (51)	0.32
12.5	63	40 (63)	41 (65)	1.00	38 (60)	35 (56)	0.72
16	9	5 (56)	4 (44)	1.00	3 (33)	3 (33)	1.00

ASHA, American Speech–Language–Hearing Association; BSA, British Society of Audiology; SBA, sound booth audiometry; TA, tablet-based audiometry.

**Table 3:** Mean pure tone thresholds per frequency (paired)

Frequency (kHz)	TA	SBA	P value	95% CI	r	95% CI	r^2^	P value
	TV (±SD) dB	TV (±SD) dB						
0.25	19.13±13.46	16.47±14.98	**<0.0001**	1.85 to 3.47	0.91	0.88 to 0.93	0.83	**<0.0001**
0.5	18.79±13.69	17.53±16.12	**0.003**	0.42 to 2.10	0.92	0.90 to 0.94	0.85	**<0.0001**
1	18.53±14.58	17.33±16.16	**0.003**	0.43 to 1.99	0.93	0.91 to 0.94	0.86	**<0.0001**
2	19.18±14.45	18.18±16.83	**0.02**	0.18 to 1.8	0.93	0.91 to 0.94	0.86	**<0.0001**
4	25.33±17.02	24.26±18.32	**0.01**	0.24 to 1.88	0.94	0.92 to 0.95	0.88	**<0.0001**
6	26.45±19.18	26.12±20.83	0.53	−0.70 to 1.36	0.93	0.90 to 0.94	0.86	**<0.0001**
8	29.95±22.38	24.95±22.37	**<0.0001**	3.89 to 6.11	0.93	0.91 to 0.95	0.86	**<0.0001**
10	29.51±22.13	31.32±22.45	0.10	−3.96 to 0.35	0.92	0.87 to 0.95	0.84	**<0.0001**
12.5	35.95±21.53	32.86±25.46	**0.003**	1.04 to 5.15	0.95	0.92 to 0.97	0.91	**<0.0001**
16	22.78±8.70	22.22±11.21	0.88	−7.92 to 9.03	0.41	−0.35 to 0.84	0.17	0.27

Bold p values are statistically significant

r2, coefficient of determination; r, Pearson Correlation coefficient; SBA, sound booth audiometry; TA, tablet-based audiometry; TV, threshold value.

Within standard measured frequencies (0.25–8 kHz) SBA had fewer unavailable results (UR) (1%, 14/1778) out of every test that could be performed, compared with TA (5%, 82/1778), where 38% (31/82) of UR were associated with high THI scores (≥38) in which tinnitus symptoms were causing moderate to severe handicap (online supplemental tables S8 and S9). At EHF (10–16 kHz), 5% (36/762) of all possible tests, using TA, were unavailable (UR), with 42% (15/36) of UR having accompanying significant tinnitus (online supplemental table S9). However, 70% (531/762) of all possible tests, using SBA, had no results available (UR) at EHF, with 85% (450/531) attributed to a lack of available EHF measuring facilities (online supplemental table S8). Non-recordable (NR) results that were beyond maximum threshold limits were greater with TA (4%, 68/1778) than SBA (1%, 17/1778) out of every test that could be performed between 0.25 and 8 kHz, which increased to 27% (202/762) and 8% (62/762), respectively, at the EHF range (online supplemental tables S1 and S10).

TA showed good sensitivity for detecting HL as defined by BSA criteria (range 77–100%) at all frequencies between 0.25 and 16 kHz and ASHA criteria (range 78–100%) between 0.25 and 12.5 kHz, with high specificity (>85%) for detecting HL using both BSA and ASHA criteria between 0.25 and 12.5 kHz (online supplemental table S11). Accuracy of TA for detecting HL was ≥88% at frequencies 0.25–12.5 kHz when assessed by both BSA and ASHA criteria. There was good PPV (≥80%) and NPV (≥81%) at all frequencies using both criteria except at 16 kHz when using ASHA criteria. Overall sensitivity, specificity, PPV, NPV and accuracy for detecting HL based on BSA and ASHA criteria using TA are shown in online supplemental table S11. There is higher sensitivity using the TA approach at EHF (8–12.5 kHz) ranging from 85% to 95% (BSA) or 78% to 100% (ASHA) compared with low frequencies (0.5–2 kHz) of 77–81% (BSA) or 80–88% (ASHA). Conversely, specificity is lower at EHF (8–12.5 kHz) between 86% and 95% (BSA) or 88% and 97% (ASHA) compared with low frequencies (0.5–2 kHz) of 97–98% (BSA) or 97–98% (ASHA).

Bland-Altman analysis (onlinesupplemental figure S2  table S12) shows that the mean differences (bias) were within 5 dB at all frequencies and above zero (except 10 kHz), with 95% limits of agreement within 15 dB of the bias between 0.25 and 6 kHz but this increased at higher frequencies (8–16 kHz). Simple linear regression was conducted to evaluate the presence of proportional bias, which identified a significant negative proportional bias for frequencies 0.25–6 and 12.5 kHz (onlinesupplemental figure S2  table S13). Using the equations generated (online supplemental table S13), online supplemental table S14 predicts the threshold when TA measurements were the same as SBA, that is, no difference between the two readings where Y=0. This was found between 25 and 30 dB for frequencies 0.5, 1, 2 and 6 kHz. There was a fixed bias observed at 8 kHz showing TA was consistently 5 dB above SBA and <2 dB lower than SBA at 10 kHz.

**Figure 1:**
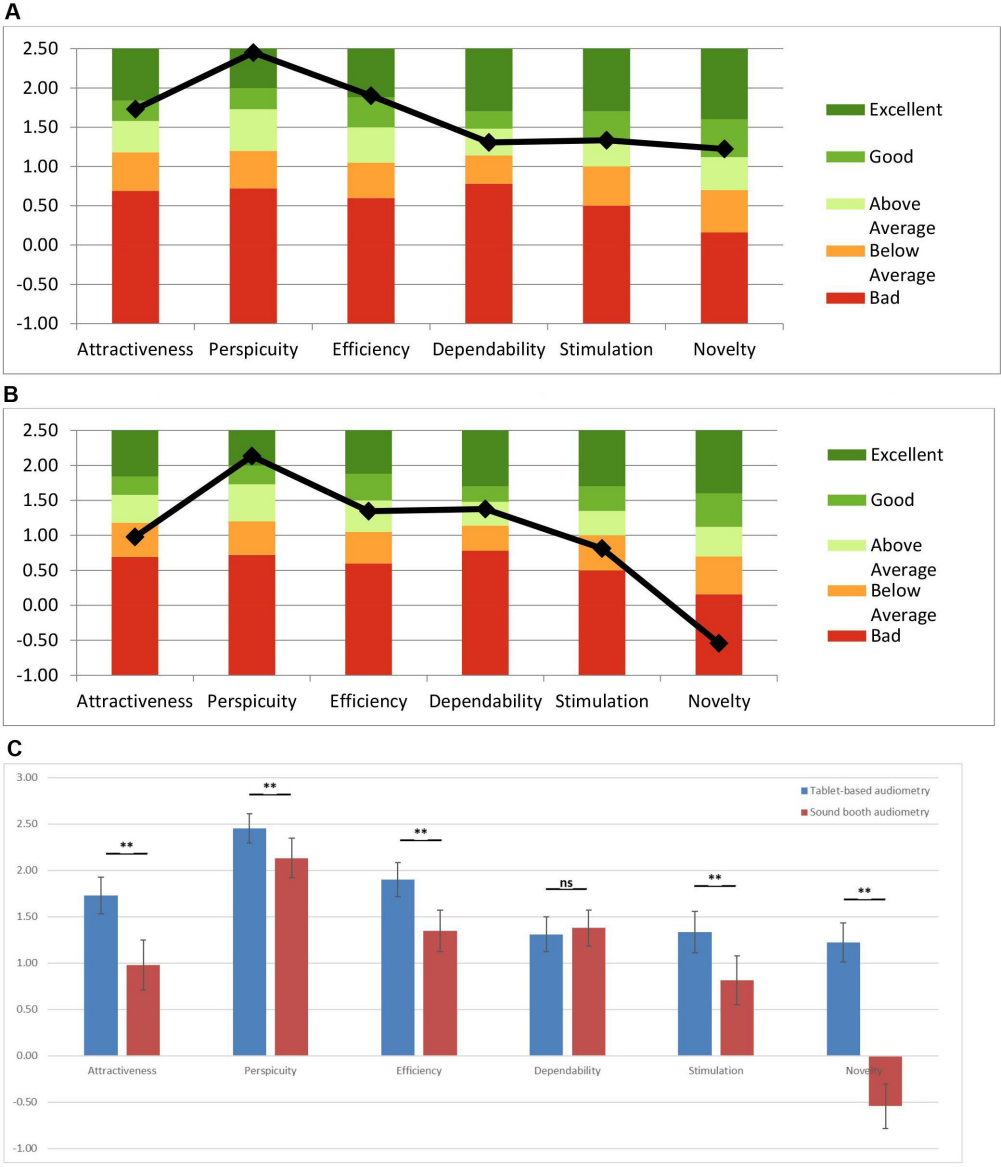
User Experience Questionnaire (UEQ) results. Comparison of scale means. (A) User Experience Questionnaire Benchmark graph of Tablet-based audiometry. (B) User Experience Questionnaire Benchmark graph for Sound-booth audiometry. (C) Comparison analysis of UEQ in tablet-based audiometry and sound booth audiometry. **Significant p<0.05. ns, not significant.

Different user processes are involved with TA (minimal human interaction with button on tablet) and SBA (audiologist and button), with the usability analysis showing that TA demonstrated good levels of attractiveness and novelty, excellent perspicuity and efficiency, and above average dependability and stimulation scales (figure 1). SBA displayed excellent perspicuity, above average efficiency and dependability, below average attractiveness and stimulation with poor novelty scales. The scale means are reported in online supplemental table S15, with Cronbach’s alpha coefficient showing acceptable reliability with all scales.

## Discussion

In this study, we present results from the largest study to date measuring the accuracy of TA to detect and screen for HL including both standard frequencies (0.25–8 kHz) and EHF (>8 kHz) ranges using circumaural headphones. TA measurements were found to be identical to SBA between 25 and 30 dB at 0.5, 1, 2 and 6 kHz, which is the threshold used to define HL according to ASHA criteria (>25 dB). TA was as effective as SBA in detecting HL for hearing thresholds between 0.25 and 12.5 kHz, regardless of whether BSA or ASHA criteria were used, with good sensitivity, specificity and accuracy. Ultimately, clinical decision-making from either method would be identical. Patient user feedback analysing usability demonstrated that TA outperformed SBA, indicating a preference for TA over SBA (as shown in Vijayasingam *et al*).^27^

Previous studies using Shoebox have shown similar agreement to SBA alongside sensitivity, specificity, PPV and NPV for HL detection to that seen within our study at standard frequency ranges.^20 21 23 24 27^ In this study, we have analysed the efficacy of TA in a cohort with a high incidence of HL (as expected in ENT/audiology outpatients) and demonstrated accuracy of HL detection according to both BSA and ASHA criteria at a wider range of frequencies. These results strongly support TA as a screening and diagnostic test to identify HL without audiometric sound booth requirement. Other mobile technologies with EHF monitoring have been used as part of ototoxicity monitoring programmes to detect chemotherapy-induced ototoxicity (OtoID, touch screen portable audiometer, HearTest using Android systems) and noise-induced HL Creare (wireless audiometer) in boothless environments such as hospital clinics and the military.^38 39^

HL is expected to rise to 2.5 billion people (1 in every 4) by 2050, due to an increase in the ageing population, with the largest increase expected in South-East Asia and Western Pacific Regions.^1^ The current provision of audiology services is insufficient to meet existing global demands, with SBA requiring audiologists, high-cost equipment and audiometric sound booths that are not always available, especially in low-resource areas.^1^ This is further compounded by the concentration of ear and hearing care (EHC) services in urban areas in many countries with limited availability in rural settings.^40 41^

Automated technology incorporated in TA allows use by non-audiologists, enabling task-sharing and reallocation with other healthcare professionals (following shorter training times) to reduce audiologist workload. This is the recommended WHO strategy to increase EHC capacity and facilitate integration of the WHO’s hearing screening and intervention; ear disease prevention and management; access to technologies; rehabilitation services; improved communication; noise reduction; greater community engagement strategy into global and national public health policy.^1^ Implementation of TA within clinical pathways has been shown to enable increased accessibility to EHC services, reducing travel barriers and waiting times in rural and urban areas and reducing current health inequalities in both LMIC and HICs.^17 42^

In this study, we identified limited facilities for EHF monitoring using SBA, with only two out of six audiometric sound booths having this capability, highlighting the limited ability for early HL detection in standard audiometric settings. TA has this provision when used with circumaural headphones, which increases accessibility to EHF monitoring and enables the potential for TA to be used to detect early changes in hearing with ototoxic chemicals/medications and noise-induced HL as part of occupational screening in workplaces and ototoxicity monitoring programmes where AG antibiotics are a core part of MDRTB, non-tuberculosis mycobacteria (NTM) and CF treatments. As demonstrated by the UEQ scores, patients found TA easier to use, more efficient, more interesting, more innovative and highlighted the usability of TA to facilitate with EHF monitoring into these hearing screening programmes. Detection of HL at early stages would help mitigate significant HL by consideration of alternative therapies or dose adjustment through shared decision-making. The portability of TA also enables use within stringent infection prevention control practices as often required in the presence of drug-resistant infections in CF, NTM or TB practices where currently routine ototoxicity monitoring is lacking.^43 44^

Digital technology such as TA could potentially improve societal HL detection if implemented in future routine hearing screening programmes increasing accessibility especially in resource-limited areas. HL is exacerbated by the time (usually 10 years) individuals accept that they have a hearing problem, with the stigma of wearing hearing aids often associated with ageing leading to delay.^45^ Additional interventions such as guidance and internet-based hearing healthcare training should accompany those recommended hearing assist devices to encourage help-seeking health behaviour.^46 47^ However, considerable challenges still remain with a secondary increased demand for aural rehabilitation services and requirement for hearing aid use. Within LMICs, the cost of hearing aids can limit uptake in individuals or services who lack the resources to purchase, fit, deliver, maintain and support hearing aid use, as well as costs of batteries and travel to EHF facilities.^48^,^50^ Health-economic modelling has suggested that increasing EHC services to cover 50% of the global population by 2030 would cost US$ 75 billion but would avert >110 million DALYs over 10 years.^1^

Our study has nevertheless highlighted some limitations in the use of TA for HL detection. Approximately one-third of individuals with UR had severe tinnitus symptoms, suggesting that SBA would be more appropriate for HL screening/detection in tinnitus patients. Although TA had a higher percentage of NR results compared with SBA, this is likely due to the shorter threshold range available, which is unlikely to affect clinical decision-making regarding hearing aid requirement (which is based on the patient’s symptomatic need and their engagement and not whether the degree of HL is severe or profound).^51^ We had fewer paired tests available at EHF in our cohort to analyse the accuracy of TA compared with SBA, given the high prevalence of HL. Furthermore, in our study, we did not monitor ambient environmental noise levels to determine if they exceeded the maximum permissible, which may explain the higher thresholds observed at low frequencies, consistently seen with other studies using mobile audiometry outside the sound booth environment.^52^ At last, this study only tested adults who were able to consent, and hence we are unable to comment on the accuracy and usability of TA in children (cognitive immaturity) and in individuals with cognitive impairment, for example, dementia.^52^

In summary, we have shown in this study that TA is an acceptable, accurate alternative to audiometric sound booth testing to increase accessibility of HL screening at standard and extended high frequency ranges and can be used as a diagnostic test for HL in individuals without significant tinnitus. Further prospective research is required to evaluate the efficacy and cost-effectiveness of TA within established clinical pathways and screening programmes. Use of TA within a global setting both in HICs and LMICs can likely assist in early HL detection, particularly where access to audiometry resources is limited.

## Supplementary material

10.1136/bmjopen-2024-097550 online supplemental file 1

10.1136/bmjopen-2024-097550 online supplemental file 2

## Data Availability

Data are available upon reasonable request.
