# Supplementary material for: Globally applicable solution to hearing loss screening: a diagnostic accuracy study of tablet-based audiometry
Source: BMJ Open. 2025 May 22;15(5):e097550. doi: 10.1136/bmjopen-2024-097550 (PMC12096992; doi:10.1136/bmjopen-2024-097550)
Supplement: online supplemental file 1 [file bmjopen-15-5-s001.docx]

**A globally applicable solution to hearing loss screening: a diagnostic accuracy study of tablet-based audiometry - Supplementary material**

**Figure S1** **Flowchart of participants**

286 Patients approached

112 declined

22 DNA

11 withdrew before consenting

12 agreed but not recruited

129 Patients consented and underwent SBA

1 patient excluded as pregnant

128 patients underwent TA

1 patient excluded SBA and TA more than one week apart

127 Patients recruited (254 ears)

Patients were tested at ten frequencies with hearing loss detection percentages reported in Table 2. SBA = sound booth audiometry, TA = Tablet-based audiometry

| Frequency | Maximum possible tests | SBA | | | TA | | | Paired  results |
| --- | --- | --- | --- | --- | --- | --- | --- | --- |
|  |  | Available results | UR | NR | Available results | UR | NR |  |
| 0.25 KHz | 254 | 251 | 2 | 1 | 233 | 12 | 9 | 231 |
| 0.5 KHz | 254 | 251 | 2 | 1 | 233 | 12 | 9 | 231 |
| 1 KHz | 254 | 251 | 2 | 1 | 234 | 11 | 9 | 232 |
| 2 KHz | 254 | 251 | 2 | 1 | 235 | 10 | 9 | 233 |
| 4 KHz | 254 | 250 | 2 | 2 | 233 | 12 | 9 | 230 |
| 6 KHz | 254 | 250 | 2 | 2 | 231 | 13 | 10 | 228 |
| 8 KHz | 254 | 243 | 2 | 9 | 229 | 12 | 13 | 222 |
| 10 KHz | 254 | 82 | 161 | 11 | 222 | 12 | 20 | 72 |
| 12.5 KHz | 254 | 77 | 156 | 21 | 196 | 12 | 46 | 63 |
| 16 KHz | 254 | 10 | 214 | 30 | 106 | 12 | 136 | 9 |

**Table S1 - Numbers of possible tests and results**

SBA = sound booth audiometry, TA = Tablet-based audiometry, KHz=Kilo Hertz, Available results (within threshold limits), UR unavailable results, NR = non recordable results as beyond maximum limits.

**Table S2 - Referrals for sound booth audiometry**

| **Referrals** | **Number of patients (%)** |
| --- | --- |
| ENT | 91 (72) |
| GP | 19 (15) |
| Hospital | 14 (11) |
| -Acute medicine | 1 |
| -Cystic fibrosis | 1 |
| -Haematology | 2 |
| -Nephrology | 1 |
| -Neurology | 3 |
| -Oncology | 6 |
| School nurse | 1 (<1) |
| Self-referral | 1 (<1) |
| (blank) | 1 (<1) |

**Table S3 - Main reason for sound booth audiometry**

| **Hearing loss** | **Number of patients (%)** |
| --- | --- |
| ENT symptoms – including (congestion, parotid gland, nasal polyps, sinusitis | 3 (2) |
| Middle ear symptoms – including cholesteatoma, otosclerosis otitis media, fungal infection, perforation, grommets, mastoiditis, retracted ear drum, eustachian tube dysfunction or congestion, conductive hearing loss | 30 (24) |
| Drug-induced (chemo/radiotherapy, COVID-19 vaccine, Kaftrio, iron chelating agent, epidural) | 5 (4) |
| Noise-induced | 2 (2) |
| Unknown | 18 (14) |
| Inner ear – including labyrinthitis, including sudden onset | 6 (5) |
| Suspected/hereditary | 2 (2) |
| Presbycusis | 1 (<1) |
| Acoustic neuroma | 1 (<1) |
| Other tumours causing hearing loss (oropharyngeal, paraglangliomas) | 2 (2) |
| Syndrome (Postural Tachycardia Syndrome, Alport, Sebastian, Turners, Susacs) | 5 (4) |
| Trauma (road traffic accident, diving) | 2 (2) |
| Dizziness/vertigo/balance | 22 (17) |
| Tinnitus | 16 (13) |
| Meniere’s disease | 1 (<1) |
| Treatment/operation protocol (chemotherapy, radiotherapy, myringoplasty) | 6 (5) |
| Auditory processing disorder | 1 (<1) |
| Ear pain | 1 (<1) |
| Impacted wax | 1 (<1) |
| Fullness in ear | 1 (<1) |
| Otitis externa | 1 (<1) |

**Table S4- Medications with ototoxic side effects (dizziness, tinnitus, other ear related)**

| **Concurrent Medications** | **N (%)** | **Medications in the previous 3 months** | **N (%)** |
| --- | --- | --- | --- |
| Antidepressants | 15 (12) | Loop diuretics | 0 (0) |
| Aspirin or NSAIDs | 11 (9) | Macrolides | 4 (3) |
| Co-trimoxazole | 1 (<1) | Intravenous aminoglycoside | 1 (<1) |
| Quinolones | 1 (<1) | Inhaled/nebulised aminoglycoside | 0 (0) |
| Tetracyclines | 1 (<1) | Ear drops containing aminoglycoside | 4 (3) |
| CFTR modulators | 1 (<1) | Vancomycin | 0 (0) |
| Bisphosphonates | 2 (2) | Cancer chemotherapy | 2 (2) |
| ACEI & A2RA | 13 (10) | Aspirin or NSAIDs | 53 (42) |
| Antiepileptics | 4 (3) | Quinine | 0 (0) |
| Opioids | 4 (3) | None | 63 (50) |
| Calcium channel blockers | 8 (6) |  |  |
| Immunosuppressants | 2 (2) |  |  |
| Antipsychotics | 2 (2) |  |  |
| Lithium | 1 (<1) |  |  |
| Atorvastatin | 7 (6) |  |  |
| Cosopt eye drops | 1 (<1) |  |  |
| Chemotherapy | 1 (<1) |  |  |
| Iron chelating agent | 1 (<1) |  |  |
| Methylphenidate | 1 (<1) |  |  |
| Rutiximab | 1 (<1) |  |  |
| None | 49 (39) |  |  |

N=number of patients, NSAIDs=non-steroidal anti-inflammatory drugs, CFTR=cystic fibrosis transmembrane conductance regulator, ACEI=angiotensin-converting enzyme inhibitors, A2RA=angiotensin 2 receptor antagonist

**Table S5 Mean pure tone thresholds per frequency with high hearing loss thresholds based according to BSA criteria (paired SBA >20dB)**

| **Frequency** |  | **TA** | **SBA** | **r** | **95% CI** | **P value** |
| --- | --- | --- | --- | --- | --- | --- |
|  | **N=** | **TV (±SD) dB** | **TV (±SD) dB** |  |  |  |
| 0∙25KHz | 56 | 36.79±16.14 | 37.95±13.97 | 0.90 | -0.7328 to 3.054 | .2245 |
| 0∙5KHz | 62 | 36.13±15.59 | 39.19±14.94 | 0.91 | 1.385 to 4.744 | **.0005** |
| 1KHz | 63 | 36.67±17.04 | 38.81±15.31 | 0.94 | 0.6305 to 3.655 | **.0062** |
| 2KHz | 70 | 36.07±15.95 | 38.64±15.67 | 0.90 | 0.8642 to 4.279 | **.0037** |
| 4KHz | 104 | 39.47±15.20 | 40.34±14.70 | 0.91 | -0.3723 to 2.103 | .1685 |
| 6KHz | 111 | 41.17±17.81 | 43.33±16.06 | 0.87 | 0.5008 to 3.824 | **.0112** |
| 8KHz | 94 | 50.27±20.06 | 46.65±16.57 | 0.88 | -5.606 to -1.628 | **.0005** |
| 10KHz | 39 | 43.59±20.52 | 47.95±16.81 | 0.91 | 1.572 to 7.146 | **.003** |
| 12∙5KHz | 41 | 48.66±15.21 | 48.9±14.38 | 0.89 | -1.987 to 2.474 | .8262 |
| 16KHz | 4 | 28.75±4.787 | 32.5±6.455 | -0.67 | -12.65 to 20.15 | .5195 |

TA = Tablet-based audiometry, SBA = sound booth audiometry, KHz=KiloHertz, TV=Threshold value, SD=standard deviation, dB=decibel, CI=confidence interval, r= Pearson Correlation coefficient, %=percentage

**Table S6 Mean pure tone thresholds per frequency with high hearing loss thresholds based according to ASHA criteria (paired SBA >25dB)**

| **Frequency** |  | **TA** | **SBA** | **R** | **95% CI** | **P value** |
| --- | --- | --- | --- | --- | --- | --- |
|  | **N=** | **TV (±SD) dB** | **TV (±SD) dB** |  |  |  |
| 0∙25KHz | 39 | 42.82±15.42 | 43.59±13.23 | 0.87 | -1.656 to 3.194 | .5247 |
| 0∙5KHz | 46 | 40.54±15.46 | 44.13±14.35 | 0.90 | 1.591 to 5.583 | **.0007** |
| 1KHz | 42 | 43.81±16.34 | 45.71±14.42 | 0.93 | -0.04048 to 3.85 | .0547 |
| 2KHz | 45 | 43.22±15.42 | 46.22±14.85 | 0.86 | 0.5736 to 5.426 | **.0166** |
| 4KHz | 84 | 42.86±14.92 | 43.99±14.07 | 0.89 | -0.3408 to 2.603 | .1302 |
| 6KHz | 91 | 44.89±17.27 | 47.36±14.97 | 0.85 | 0.5754 to 4.370 | **.0112** |
| 8KHz | 83 | 52.95±19.49 | 49.52±15.49 | 0.87 | -5.535 to -1.332 | **.0017** |
| 10KHz | 37 | 45.14±19.88 | 49.19±16.35 | 0.90 | 1.171 to 6.937 | **.0072** |
| 12∙5KHz | 35 | 52.86±11.96 | 53±11.19 | 0.80 | -2.373 to 2.659 | .9088 |
| 16KHz | 3 | 26.67±2.887 | 35±5.00 | 0.00 | -6.009 to 22.68 | .1296 |

TA = Tablet-based audiometry, SBA = sound booth audiometry, KHz=KiloHertz, TV=Threshold value, SD=standard deviation, dB=decibel, CI=confidence interval, r= Pearson Correlation coefficient, %=percentage

**Table S7– Tablet audiometry threshold difference within 10dB of Sound Booth Audiometry**

| **Frequency** | **Paired results** | **Paired results within 10dB of SBA (%)** |
| --- | --- | --- |
| 0.25KHz | 231 | 216 (94) |
| 0.5KHz | 231 | 220 (95) |
| 1KHz | 232 | 225 (97) |
| 2KHz | 233 | 221 (95) |
| 4KHz | 230 | 222 (97) |
| 6KHz | 228 | 207 (91) |
| 8KHz | 222 | 178 (80) |
| 10KHz | 72 | 63 (88) |
| 12.5KHz | 63 | 53 (84) |
| 16KHz | 9 | 7 (78) |
| Total | 1751 | 1612 (92) |

KHz=KiloHertz, SBA = sound booth audiometry, dB=decibel

**Table S8- Sound booth audiometry unavailable results (UR)**

|  | **Sound booth audiometry Unavailable results, N (%)** | | | |
| --- | --- | --- | --- | --- |
|  | 0.25-8KHz | 10KHz | 12.5KHz | 16KHz |
| Digital failure - Lost results | 14 (100) | 2 (1) | 2 (1) | 2 (1) |
| Digital failure - 10-16KHz not available |  | 2 (1) | 2 (1) | 2 (1) |
| 10-16KHz not requested |  | 2 (1) | 2 (1) | 2 (1) |
| Not measured – unknown |  | 5 (3) |  | 58 (27) |
| 10-16KHz Not available |  | 150 (93) | 150 (96) | 150 (70) |
| Total | 14 | 161 | 156 | 214 |

KHz=KiloHertz

**Table S9–Tablet-based audiometry unavailable results (UR)**

|  | **Tablet-based audiometry unavailable results, N (%)** | | | | | | | | | |
| --- | --- | --- | --- | --- | --- | --- | --- | --- | --- | --- |
|  | 0.25KHz | 0.5KHz | 1KHz | 2KHz | 4KHz | 6KHz | 8KHz | 10KHz | 12.5KHz | 16KHz |
| THI=0-16 | 0 | 0 | 0 | 0 | 0 | 1 (8) | 0 | 0 | 0 | 0 |
| THI=38-56 | 2 (17) | 2 (17) | 1 (9) | 2 (20) | 2 (17) | 2 (15) | 2 (17) | 2 (17) | 2 (17) | 2 (17) |
| THI=58-76 | 3 (25) | 3 (25) | 1 (9) | 2 (20) | 3 (25) | 3 (23) | 3 (25) | 3 (25) | 3 (25) | 3 (25) |
| Upload failure | 4 (33) | 4 (33) | 4 (36) | 4 (40) | 4 (33) | 4 (31) | 4 (33) | 4 (33) | 4 (33) | 4 (33) |
| Unknown | 3 (25) | 3 (25) | 5 (45) | 2 (20) | 3 (25) | 3 (23) | 3 (25) | 3 (25) | 3 (25) | 3 (25) |
| Total | 12 | 12 | 11 | 10 | 12 | 13 | 12 | 12 | 12 | 12 |

THI=Tinnitus Handicap Inventory, KHz=KiloHertz,

**Table S10 - Threshold Limits**

| **Frequency** | **Tablet-based audiometry** | | **Sound booth audiometry** | | | |
| --- | --- | --- | --- | --- | --- | --- |
|  | Minimum dB | Maximum dB | Minimum dB | | Maximum dB | |
|  |  |  | GST | UHD | GST | UHD |
| 0.25KHz | 10 | 90 | -10 | -10 | 90 | 105 |
| 0.5KHz | 10 | 90 | -10 | -10 | 110 | 110 |
| 1KHz | 10 | 90 | -10 | -10 | 110 | 110 |
| 2KHz | 10 | 90 | -10 | -10 | 110 | 110 |
| 4KHz | 10 | 90 | -10 | -10 | 110 | 110 |
| 6KHz | 10 | 90 | -10 | -10 | 100 | 110 |
| 8KHz | 10 | 90 | -10 | -10 | 70 | 105 |
| 10KHz | 10 | 85 | -20 | - | 80 | - |
| 12.5KHz | 10 | 80 | -20 | - | 70 | - |
| 16KHz | 10 | 55 | -20 | - | 40 | - |

dB=decibel, KHz=KiloHertz, GST=Guy’s & St Thomas’, UHD=University Hospitals Dorset

**Table S11 Tablet-based audiometry sensitivity and specificity for hearing loss detection according to BSA and ASHA criteria**

| Frequency | N | BSA >20dB, % (95% CI) | | | | | ASHA >25dB, % (95% CI) | | | | |
| --- | --- | --- | --- | --- | --- | --- | --- | --- | --- | --- | --- |
|  |  | Sensitivity | Specificity | PPV | NPV | Accuracy | Sensitivity | Specificity | PPV | NPV | Accuracy |
| 0∙25KHz | 231 | 79 (66-88) | 94 (90-97) | 81 (70-89) | 93 (89-96) | 90 (86-94) | 82 (66-92) | 96 (92-98) | 80 (67-89) | 96 (93-98) | 94 (90-96) |
| 0∙5KHz | 231 | 77 (65-87) | 97 (93-99) | 91 (80-96) | 92 (88-95) | 92 (87-95) | 80 (66-91) | 98 (95-99) | 90 (78-96) | 95 (92-97) | 94 (91-97) |
| 1KHz | 232 | 81 (69-90) | 97 (93-99) | 91 (81-96) | 93 (89-96) | 93 (89-96) | 88 (74-96) | 98 (95-100) | 93 (80-97) | 97 (94-99) | 97 (93-99) |
| 2KHz | 233 | 81 (70-90) | 98 (94-99) | 93 (84-97) | 92 (88-95) | 93 (89-96) | 84 (71-94) | 97 (93-99) | 86 (74-93) | 96 (93-98) | 94 (91-97) |
| 4KHz | 230 | 93 (87-97) | 92 (86-96) | 91 (84-95) | 94 (89-97) | 93 (88-96) | 88 (79-94) | 92 (87-96) | 87 (79-92) | 93 (88-96) | 91 (86-94) |
| 6KHz | 228 | 84 (76-90) | 98 (94-100) | 98 (92-99) | 86 (81-91) | 91 (87-95) | 85 (76-91) | 95 (90-98) | 92 (84-96) | 90 (85-94) | 91 (86-94) |
| 8KHz | 222 | 90 (83-96) | 86 (79-91) | 83 (75-88) | 92 (87-96) | 88 (83-92) | 89 (80-95) | 88 (81-93) | 81 (73-87) | 93 (88-96) | 88 (83-92) |
| 10KHz | 72 | 85 (69-94) | 91 (76-98) | 92 (79-97) | 83 (70-91) | 88 (78-94) | 78 (62-90) | 97 (85-100) | 97 (81-100) | 81 (70-89) | 88 (78-94) |
| 12∙5KHz | 63 | 95 (83-99) | 95 (77-100) | 98 (85-100) | 91 (73-98) | 95 (87-99) | 100 (90-100) | 89 (72-98) | 92 (80-97) | 100 (86-100) | 95 (87-99) |
| 16KHz | 9 | 100 (40-100) | 80 (28-99) | 80 (41-96) | 100 (40-100) | 89 (52-100) | 33 (1-91) | 67 (22-96) | 33 (7-78) | 67 (43-84) | 56 (21-86) |
| Overall 0∙25-16KHz | 1751 | 86 (83-88) | 95 (93-96) | 90 (88-92) | 92 (90-93) | 91 (90-93) | 86 (83-89) | 95 (94-96) | 88 (85-90) | 94 (93-95) | 92 (91-94) |
| Overall 0∙25-8KHz | 1607 | 85 (82-88) | 95 (93-96) | 90 (87-92) | 92 (91-93) | 91 (90-93) | 86 (82-89) | 95 (94-96) | 87 (84-90) | 95 (94-96) | 93 (91-94) |
| Overall 10-16KHz | 144 | 90 (82-96) | 92 (82-97) | 94 (87-97) | 87 (78-93) | 91 (85-95) | 87 (77-93) | 91 (82-97) | 92 (83-96) | 86 (78-92) | 89 (83-94) |

N=paired results, BSA= British Society of Audiology, ASHA= American Speech-Language-Hearing Association, dB=decibel, KHz=KiloHertz, CI=confidence interval, PPV= Positive predictive value, NPV=Negative predictive value

**Table S12 - Bland Altman results for each frequency**

| Frequency | N= | Bias | SD of Bias | 95% limits of agreement | |
| --- | --- | --- | --- | --- | --- |
|  |  |  |  | From | To |
| 0.25KHz | 231 | 2.662 | 6.251 | -9.590 | 14.91 |
| 0.5KHz | 231 | 1.255 | 6.439 | -11.37 | 13.88 |
| 1KHz | 232 | 1.207 | 6.035 | -10.62 | 13.03 |
| 2KHz | 233 | 1.009 | 6.395 | -11.53 | 13.54 |
| 4KHz | 230 | 1.065 | 6.291 | -11.27 | 13.40 |
| 6KHz | 228 | 0.3289 | 7.867 | -15.09 | 15.75 |
| 8KHz | 222 | 5.000 | 8.388 | -11.44 | 21.44 |
| 10KHz | 72 | -1.806 | 9.166 | -19.77 | 16.16 |
| 12.5KHz | 63 | 3.095 | 8.153 | -12.89 | 19.08 |
| 16KHz | 9 | 0.5556 | 11.02 | -21.05 | 22.16 |

N=paired results, KHz=KiloHertz, SD= standard deviation

**Table S13 - Simple linear regression**

| **Frequency** | **r^2^** | **95% CI** | **P value** | **F statistic** | **DFn, DFd** | **Equation** |
| --- | --- | --- | --- | --- | --- | --- |
| 0.25KHz | 0.06170 | -0.1685 to -0.05501 | **0.0001** | 15.06 | 1, 229 | Y = -0.1118*X + 4.652 |
| 0.5KHz | 0.1479 | -0.2226 to -0.1166 | **<0.0001** | 39.74 | 1, 229 | Y = -0.1696*X + 4.335 |
| 1KHz | 0.07137 | -0.1569 to -0.05676 | **<0.0001** | 17.68 | 1, 230 | Y = -0.1068*X + 3.122 |
| 2KHz | 0.1434 | -0.2077 to -0.1078 | **<0.0001** | 38.68 | 1, 231 | Y = -0.1577*X + 3.955 |
| 4KHz | 0.04449 | -0.1224 to -0.03014 | **0.0013** | 10.61 | 1, 228 | Y = -0.07627*X + 2.956 |
| 6KHz | 0.04556 | -0.1369 to -0.03422 | **0.0012** | 10.79 | 1, 226 | Y = -0.08553*X + 2.577 |
| 8KHz | 1.506e-006 | -0.05024 to 0.05118 | 0.9855 | 0.0003313 | 1, 220 | Y = 0.0004683*X + 4.987 |
| 10KHz | 0.001346 | -0.1155 to 0.08468 | 0.7596 | 0.09434 | 1, 70 | Y = -0.01541*X - 1.337 |
| 12.5KHz | 0.2376 | -0.2496 to -0.09264 | **<0.0001** | 19.01 | 1, 61 | Y = -0.1711*X + 8.983 |
| 16KHz | 0.07314 | -1.487 to 0.7756 | 0.4815 | 0.5524 | 1, 7 | Y = -0.3556*X + 8.556 |

r^2^= coefficient of determination, CI= confidence interval, DFn= numerator degrees of freedom (regression df), DFd = denominator degrees of freedom (residual df), F statistic= explained variance Unexplained variance.

**Table S14 Threshold at which Tablet-based Audiometry and Sound Booth Audiometry are equal**

| **Frequency** | **Threshold (dB)** |
| --- | --- |
| 0.25KHz | 41.6 |
| 0.5KHz | 25.6 |
| 1KHz | 29.2 |
| 2KHz | 25.1 |
| 4KHz | 38.8 |
| 6KHz | 30.1 |
| 8KHz | N/A |
| 10KHz | N/A |
| 12.5KHz | -52.5 |
| 16KHz | N/A |

dB=decibel, KHz=KiloHertz, N/A=not applicable

**Table S15 - User Experience Questionnaire (UEQ) results of tablet-based audiometry & sound booth audiometry**

1. **Tablet-based audiometry**

| **Scale** | **Mean** | **Std. Dev.** | **N** | **Confidence** | **Confidence interval** | | **Cronbachs Alpha-coefficient** | **Confidence interval Cronbachs Alpha** |
| --- | --- | --- | --- | --- | --- | --- | --- | --- |
| **Attractiveness** | 1.729 | 0.998 | 100 | 0.196 | 1.533 | 1.924 | 0.85 | 0.80-0.89 |
| **Perspicuity** | 2.450 | 0.808 | 100 | 0.158 | 2.292 | 2.608 | 0.75 | 0.65-0.62 |
| **Efficiency** | 1.900 | 0.951 | 100 | 0.186 | 1.714 | 2.086 | 0.69 | 0.56-0.77 |
| **Dependability** | 1.308 | 0.957 | 100 | 0.188 | 1.120 | 1.495 | 0.57 | 0.41-0.69 |
| **Stimulation** | 1.335 | 1.136 | 100 | 0.223 | 1.112 | 1.558 | 0.82 | 0.75-0.87 |
| **Novelty** | 1.223 | 1.073 | 100 | 0.210 | 1.012 | 1.433 | 0.63 | 0.49-0.73 |

1. **Sound booth audiometry**

| **Scale** | **Mean** | **Std. Dev.** | **N** | **Confidence** | **Confidence interval** | | **Cronbachs Alpha coefficient** | **Confidence interval Cronbachs Alpha** |
| --- | --- | --- | --- | --- | --- | --- | --- | --- |
| **Attractiveness** | 0.977 | 1.324 | 93 | 0.269 | 0.708 | 1.247 | 0.91 | 0.88-0.94 |
| **Perspicuity** | 2.132 | 1.051 | 93 | 0.214 | 1.918 | 2.345 | 0.87 | 0.82-0.91 |
| **Efficiency** | 1.347 | 1.113 | 93 | 0.226 | 1.121 | 1.573 | 0.68 | 0.56-0.77 |
| **Dependability** | 1.376 | 0.945 | 93 | 0.192 | 1.184 | 1.568 | 0.56 | 0.39-0.69 |
| **Stimulation** | 0.812 | 1.296 | 93 | 0.263 | 0.548 | 1.075 | 0.86 | 0.81-0.90 |
| **Novelty** | -0.543 | 1.188 | 93 | 0.241 | -0.784 | -0.302 | 0.69 | 0.57-0.78 |

1. **T-test of scale means tablet-based audiometry compared with sound booth audiometry**

| **Scale** | **P value** |
| --- | --- |
| Attractiveness | <0.0001 |
| Perspicuity | 0.0201 |
| Efficiency | 0.0003 |
| Dependability | 0.6158 |
| Stimulation | 0.0033 |
| Novelty | <0.0001 |
