# Supplementary material for: Globally applicable solution to hearing loss screening: a diagnostic accuracy study of tablet-based audiometry
Source: BMJ Open. 2025 May 22;15(5):e097550. doi: 10.1136/bmjopen-2024-097550 (PMC12096992; doi:10.1136/bmjopen-2024-097550)

Figure S2 Bland Altman plot per frequency: difference between tablet-based and sound booth audiometry verses average hearing threshold

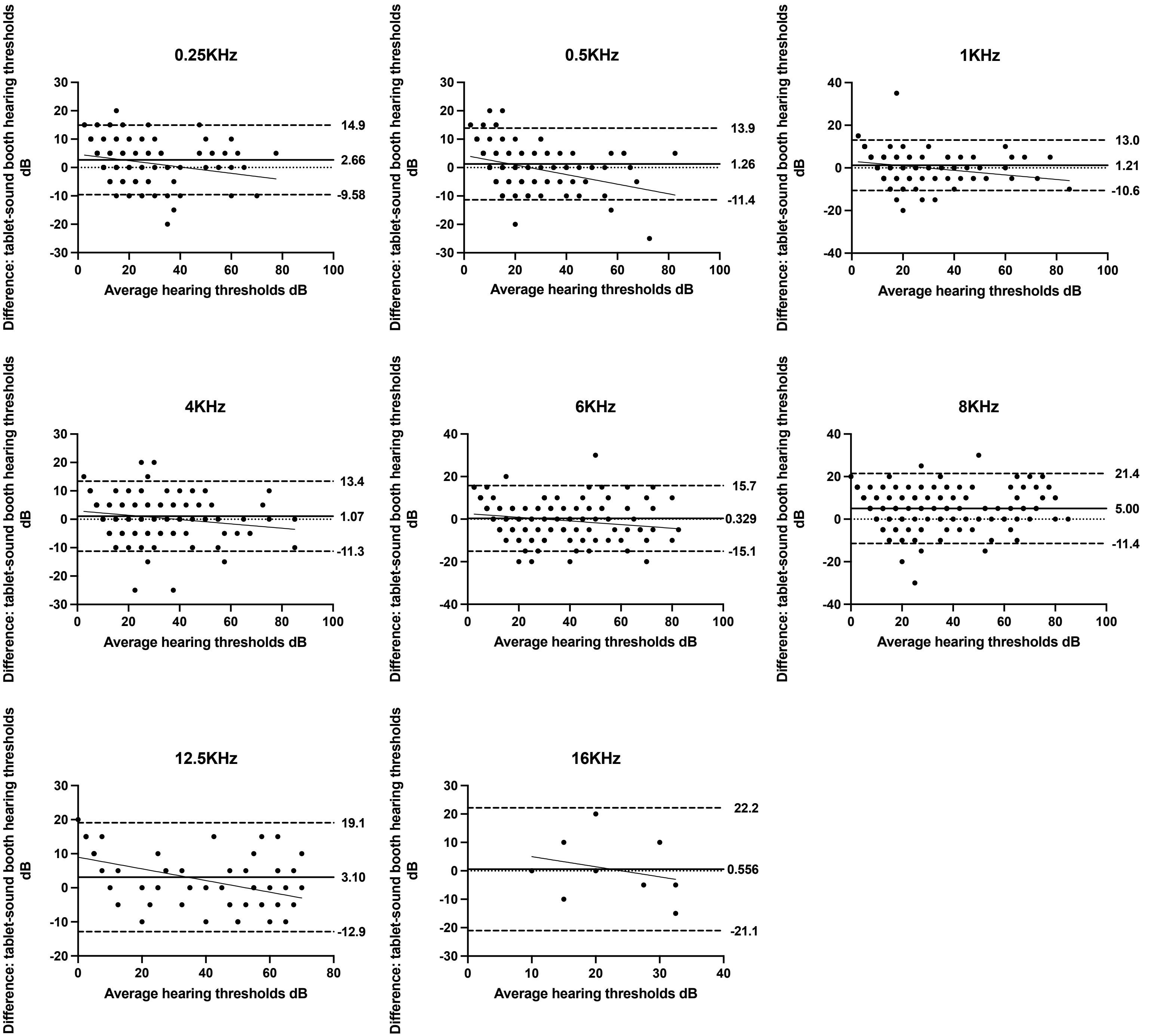

Supplement: online supplemental file 2 [file bmjopen-15-5-s002.pdf]
