# Supplementary material for: Globally applicable solution to hearing loss screening: a diagnostic accuracy study of tablet-based audiometry
Source: BMJ Open. 2025 May 22;15(5):e097550. doi: 10.1136/bmjopen-2024-097550 (PMC12096992; doi:10.1136/bmjopen-2024-097550)
Supplement: Supplementary file 3 [file bmjopen-2024-097550.reviewer_comments.pdf]

PEER REVIEW HISTORY

BMJ Open publishes all reviews undertaken for accepted manuscripts. Reviewers are asked to complete a checklist review form and are provided with free text boxes to elaborate on their assessment. These free text comments are reproduced below.

ARTICLE DETAILS

Title (Provisional)

A globally applicable solution to hearing loss screening: a diagnostic accuracy study of tablet-based audiometry

Authors

Cheong, Jamie; Lowe, Emily; Lee, Chang Woo; Barbosa, Claudia; Gillen, Lise; King, Emma; Premachandra, Presanna; Shah, Anand; Drobniewski, Francis

---

VERSION 1 - REVIEW

**Reviewer**	**1**
**Name**	**Košec, A**
**Affiliation**	**University of Zagreb School of Medicine, Otorhinolaryngology and Head&Neck Surgery**
**Date**	**02-Feb-2025**
**COI**	**None**

The title and abstract are fine. The paper deals with the question whether tablet based audiometry may be considered equivalent to sound booth audiometry.

The introduction gives enough background information on hearing measurements, hearing loss and relevance of accurate measurements. The aims are clearly stated.

The MM section list complete inclusion and exclusion criteria, with main outcome measures being PTA hearing thresholds between the golden standard and a novel hearing test technique.

Type of study - cross-sectional comparative observational needs to be listed, as well as STROBE guidelines and flowchart.

IRB consent is noted, as well as trial registration. Statistics were adequate. The results display the main audiometric outcomes and give a honest analysis of the results. The discussion highlights the use of tablet audiometry as a possible screening measure and I have no issues with the study's internal or external validity. Limitations were discussed in detail.

I would support publishing the results pending minor revision.

Reviewer 2

Name Joshi, Deepashree

Affiliation Sri Ramachandra Institute of Higher Education and Research, Faculty of Audiology and Speech Language Pathology

Date 25-Feb-2025

COI None

I appreciate the opportunity to review this article. The study is well planned, ordered, and organized as a whole. My comments for the author are

Introduction

The paragraph beginning with "Impact of unaddressed..." at line 39 does not align with the preceding paragraph.

Requirement for boothless EFA is not emphasized in the introduction.

Several additional studies have using shoebox for hearing screening and assessed its accuracy. It is essential to cite them in the introduction, specifying how they differ from the present study and justifying the necessity for the current research.

Method

Does this study focus solely on the validation of EFA in Shoebox, in addition to the pre-existing validity values?

To enhance the method's consistency and comprehension, the following considerations may be made.

On page 5, The last line of the paragraph, "Demographic information...", can be moved to the first paragraph of the method as follows: "Audiometry and data collection were performed prospectively in this diagnostic accuracy study of the Shoebox Standard Limited portable audiometer compared to SBA as the gold standard." Patients were recruited from audiology and ENT outpatient clinics at Guy’s & St Thomas’ NHS Foundation Trust and University Hospitals Dorset NHS Foundation Trust across two locations in the UK. …….. Ethical approval was obtained from the Health Research Authority (HRA) and Health and Care Research Wales (HCRW) West Midlands – Edgbaston Research Ethics Committee on February 4, 2022 (IRAS project ID 298372) and registered on clinicaltrials.gov (NCT05847556).

Following that, the second paragraph may begin with demographic information and the process used to collect case history, among other things.

The terms "patient" and "participant" are used interchangeably throughout the methodology. Is there any particular reason for that i.e., the participants are considered as

individuals with normal hearing and patients are considered as individuals with hearing impairment? How were the inclusion criteria established for normal hearing and hearing impairment? If not, please use one term consistently.

The methodology lacks clarity about the inclusion criteria for participants, except than age. Were those with exposure to ototoxic medications and noise included? If so, how was this information collected?

On page 4, in the second paragraph of the methods section, the authors mention the 'User Experience Questionnaire.' I recommend that the authors include further details regarding the questionnaire, including the total number of questions it contains.What are the domains or categories of the tool? Is it gathered in a paper-pencil format or a computerized survey form? Details regarding the rating scale? Generally, I would suggest the author to add a procedure for administration of questionnaire.

In the same paragraph, the justification for using HHI, THI, and DHI in a diagnostic accuracy research may be included.

What was the duration required to complete data collection for one participant, considering that the procedure included two tests and the administration of questionnaires?

Results

I believe that the analysis about ethnicity is unnecessary and recommend that the authors skip that section.

In Table 3, Mean pure tone thresholds are analyzed. Nonetheless, I observed that these mean values pertain to the entire number of participants for each frequency. Is it feasible to do a sub-analysis by exclusively comparing individuals with high thresholds (hearing loss) at each frequency (>25 dB)? It will provide insight into the suitability of the TA relative to the SBA in identifying elevated thresholds.

Table 4 and the third paragraph on page 8, beginning with “TA showed…,” demonstrate the sensitivity and specificity of TA in relation to SBA across various frequency thresholds. I recommend that the author include sensitivity and specificity values for the following.

1. Comparison of Standard Pure Tone Averages (500 Hz, 1 kHz, and 2 kHz) in TA and SBA.

2. Comparison of High Frequency Averages (8 kHz, 10 kHz, 12.5 kHz) in TA and SBA.

On page 8, in the results section, how did the authors compare the outcomes of the User Experience Questionnaire? It is explicitly noted in the procedure that TA was automated and conducted by the participants themselves, while SBA was performed by an audiologist. Consequently, the participants would have lacked familiarity with the SBA, rendering a comparison of the user experiences between TA and SBA unsuitable. Given that the users were entirely different. For TA, the participants themselves were the users, whereas for SBA, the users were audiologists.

I recommend that the authors eliminate the comparison section (Figures B and C) from Table S1 (B and C). Instead, include further details regarding the user experience questionnaire items and the scoring criteria for each item. Which item score contributes to the aggregate scale score, etc.

Discussion

On discussion page 11, the paragraph commencing with "although the use of..." is lengthy and elaborates on the shortage in hearing aid usage and its awareness. The paragraph can emphasize how the efficacy of hearing aid usage may be enhanced in the near future with the implementation of TA-based routine hearing screening programs.

The discussion may also encompass a comparison of Shoebox TA with any other currently available tablet-based hearing screener for adults that addresses extended high frequencies, such as the study by Brungart et al. (2017). Using tablet-based technology to deliver time-efficient ototoxicity monitoring. International Journal of Audiology, 57(sup4), S78–S86. https://doi.org/10.1080/14992027.2017.1370138

Ehlert K, Heinze B, Graham MA, Swanepoel D. Surveillance for ototoxicity in platinum-based chemotherapy using mobile health audiometry with extended high frequencies. The Journal of Laryngology & Otology, 2023; 137(1): 61-67. doi:10.1017/S0022215122001281

Another unique aspect of the paper is the inclusion of a user experience survey. Nevertheless, the findings were not addressed in the discussion. I recommend that the authors explore the results of the user experience survey and how it demonstrates the potential of the ShoeBox TA in facilitating the implementation of automated tablet-based EHF hearing screening programs for monitoring noise exposure and ototoxicity.

VERSION 1 - AUTHOR RESPONSE

Reviewer: 1
Dr. A Košec, University of Zagreb School of Medicine
Comments to the Author:
The title and abstract are fine. The paper deals with the question whether tablet based audiometry may be considered equivalent to sound booth audiometry.
The introduction gives enough background information on hearing measurements, hearing loss and relevance of accurate measurements. The aims are clearly stated.
The MM section list complete inclusion and exclusion criteria, with main outcome measures being PTA hearing thresholds between the golden standard and a novel hearing test technique.
Type of study - cross-sectional comparative observational needs to be listed, as well as STROBE guidelines and flowchart.

Response

Thank you for your comments. The STARD guidelines were followed as this is a diagnostic accuracy study and the STARD checklist was submitted with the manuscript. These guidelines have been added in the methods and referenced in the methods section to clarify which guidelines were adhered to:

‘Audiometry and data collection were performed prospectively in this diagnostic accuracy study conforming to STARD guidelines of the Shoebox Standard Limited portable audiometer compared to SBA as the gold standard.(28)’

Cross-sectional comparative observational study and STROBE guidelines have therefore not been added. A flow chart has been added to the supplementary material as Figure S1.

IRB consent is noted, as well as trial registration. Statistics were adequate. The results display the main audiometric outcomes and give a honest analysis of the results. The discussion highlights the use of tablet audiometry as a possible screening measure and I have no issues with the study's internal or external validity. Limitations were discussed in detail.
I would support publishing the results pending minor revision.

Reviewer: 2
Dr. Deepashree Joshi, Sri Ramachandra Institute of Higher Education and Research
Comments to the Author:
I appreciate the opportunity to review this article. The study is well planned, ordered, and organized as a whole. My comments for the author are

Introduction

The paragraph beginning with "Impact of unaddressed..." at line 39 does not align with the preceding paragraph.

Response

The first sentence of the 2nd paragraph in the introduction has been rephrased to improve alignment with the 1st paragraph. The end of the previous paragraph has been included to give context:

‘Despite their widespread use, systems for identifying early HL in low- or middle-income countries (LMICs) are scarce.(5)

Delayed diagnosis of HL in children can cause speech and language development issues continuing into adulthood, with children less likely to go onto higher education, more likely to be unemployed, have poorer mental health, lower quality of life, and social isolation affecting cognition.’

Requirement for boothless EFA is not emphasized in the introduction.

Response

The use and value of boothless EHA has been emphasised in the introduction:

‘Boothless audiometry measuring EHF is therefore required to detect HL in ototoxic drug and noise exposure or where SBA is unavailable.’

Several additional studies have using shoebox for hearing screening and assessed its accuracy. It is essential to cite them in the introduction, specifying how they differ from the present study and justifying the necessity for the current research.
Response:

Thank you for your comments. Previous studies using Shoebox as TA in accuracy studies have been cited in the introduction, (references 19-26) with specifics about how they differ from current study, with justification for current research added:

'TA using Shoebox has been validated in other studies in children and adults attending audiology outpatients, emergency departments, or patients with cognitive impairment, at frequencies up to 8KHz.(19–26). Our previous work has demonstrated the use of TA as an accurate screening tool in individuals with cystic fibrosis (CF) up to frequencies of 12.5KHz.(27) Unlike most other published studies, we have conducted a multicentre study, analysing TA accuracy compared to SBA at both standard frequencies (0.25-8KHz) and EHF up to 16KHz, in a general audiology and ear, nose and throat (ENT) outpatient clinic setting. Additionally, we have also focused on assessing the TA and SBA approaches from the patients' perspective, including the usability of both audiometric processes.'

19. Yeung J, Javidnia H, Heley S, Beauregard Y, Champagne S, Bromwich M. The new age of play audiometry: Prospective validation testing of an iPad-based play audiometer. Journal of Otolaryngology - Head and Neck Surgery. 2013;42(MAR):1–7.

20. Yeung JC, Heley S, Beauregard Y, Champagne S, Bromwich MA. Self-administered hearing loss screening using an interactive, tablet play audiometer with ear bud headphones. Int J Pediatr Otorhinolaryngol. 2015;79(8):1248–52.

21. Thompson GP, Sladen DP, Borst BJH, Still OL. Accuracy of a Tablet Audiometer for Measuring Behavioral Hearing Thresholds in a Clinical Population. Otolaryngology - Head and Neck Surgery (United States). 2015;153(5):838–42.

22. Saliba J, Al-Reefi M, Carriere JS, Verma N, Provencal C, Rappaport JM. Accuracy of Mobile-Based Audiometry in the Evaluation of Hearing Loss in Quiet and Noisy Environments. Otolaryngology - Head and Neck Surgery (United States). 2017 Apr 1;156(4):706–11.

23. Lubner RJ, Barbarite E, Kondamuri N, Knoll RM, Ota HG, Lewis RM, et al. Hearing Vital Signs: Mobile Audiometry in the Emergency Department for Evaluation of Sudden Hearing Loss. Otolaryngology - Head and Neck Surgery (United States). 2020 Nov 1;163(5):1025–8.

24. Garcia A, Chari DA, Stankovic KM, Lee DJ, Kozin ED, Franck KH. Implementation of Mobile Audiometry During the COVID-19 Pandemic. Otolaryngology - Head and Neck Surgery (United States). 2022 Sep 1;167(3):465–8.

25. Frank A, Goldlist S, Mark Fraser AE, Bromwich M. Validation of SHOEBOX QuickTest Hearing Loss Screening Tool in Individuals With Cognitive Impairment. Front Digit Health. 2021 Sep 13;3.

26. Bastianelli M, Mark AE, McAfee A, Schramm D, Lefrançois R, Bromwich M. Adult validation of a self-administered tablet audiometer. Journal of Otolaryngology - Head and Neck Surgery. 2019 Nov 7;48(1).

Method

Does this study focus solely on the validation of EFA in Shoebox, in addition to the pre-existing validity values?

Response

We validated both standard frequencies 0.25-8KHz as well as EHF (10-16KHz) in a cohort of adult audiology and ENT patients. In addition, we analysed patient perspectives and assessed the usability

of tablet-based audiometry in an ENT and audiology clinic setting. We have amended the last line of the introduction (which is also in the previous response) to say:

'Unlike most other published studies, we have conducted a multicentre study, analysing TA accuracy compared to SBA at both standard frequencies (0.25-8KHz) and EHF up to 16KHz, in a general audiology and ear, nose and throat (ENT) outpatient clinic setting. Additionally, we have also focused on assessing the TA and SBA approaches from the patients' perspective, including the usability of both audiometric processes.'

To enhance the method's consistency and comprehension, the following considerations may be made.
On page 5, The last line of the paragraph, "Demographic information...", can be moved to the first paragraph of the method as follows: "Audiometry and data collection were performed prospectively in this diagnostic accuracy study of the Shoebox Standard Limited portable audiometer compared to SBA as the gold standard." Patients were recruited from audiology and ENT outpatient clinics at Guy's & St Thomas' NHS Foundation Trust and University Hospitals Dorset NHS Foundation Trust across two locations in the UK. …….. Ethical approval was obtained from the Health Research Authority (HRA) and Health and Care Research Wales (HCRW) West Midlands – Edgbaston Research Ethics Committee on February 4, 2022 (IRAS project ID 298372) and registered on clinicaltrials.gov (NCT05847556).

Following that, the second paragraph may begin with demographic information and the process used to collect case history, among other things.

Response

Many thanks. The suggestions above have been incorporated into the manuscript:

'Audiometry and data collection were performed prospectively in this diagnostic accuracy study conforming to STARD guidelines of the Shoebox Standard Limited portable audiometer compared to SBA as the gold standard.(28) Patients were recruited from audiology and ENT outpatient clinics at Guy's & St Thomas' NHS Foundation Trust and University Hospitals Dorset NHS Foundation Trust across two locations in the UK. Ethical approval was obtained from the Health Research Authority (HRA) and Health and Care Research Wales (HCRW) West Midlands – Edgbaston Research Ethics Committee on February 4, 2022 (IRAS project ID 298372) and registered on clinicaltrials.gov (NCT05847556) where the full protocol can be accessed.

Demographic information, medical and drug history, referral, reason for audiometry if known were recorded. Data were entered onto a RedCap database (14·1·4). Definition of HL was defined by the British Society of Audiology (BSA) as thresholds >20dB and American Speech-Language-Hearing Association (ASHA) standards as thresholds >25dB.(15,29) Scores for hearing-related questionnaires were assigned: no (0 points), sometimes (2 points) and yes (4 points) with total scores categorised into different severities.(30–32)'

The terms "patient" and "participant" are used interchangeably throughout the methodology. Is there any particular reason for that i.e., the participants are considered as individuals with normal hearing and patients are considered as individuals with hearing impairment? How were the inclusion criteria established for normal hearing and hearing impairment? If not, please use one term consistently.

Response

Participant has been replaced with patient so one term is used consistently.

The methodology lacks clarity about the inclusion criteria for participants, except than age. Were those with exposure to ototoxic medications and noise included? If so, how was this information collected?

Response

The following has been added to clarify the above:

'Any patient aged 16 years or over attending the ENT and audiology clinics, between April 2022 and September 2023, who consented were eligible to participate. Those exposed to ototoxic medicines or noise and who agreed to take part in the study were also included and listed in the Supplementary material.'

On page 4, in the second paragraph of the methods section, the authors mention the 'User Experience Questionnaire.' I recommend that the authors include further details regarding the questionnaire, including the total number of questions it contains.What are the domains or categories of the tool? Is it gathered in a paper-pencil format or a computerized survey form? Details regarding the rating scale? Generally, I would suggest the author to add a procedure for administration of questionnaire.

Response

The following has been added to the methods to expand on the details about the UEQ:

'The UEQ assesses hedonic and pragmatic quality aspects involving 26 polarised statements graded on a 7-point Likert scale to gauge patient opinions on six scales: attractiveness, perspicuity, dependability, efficiency, novelty, stimulation. This was created on a Qualtrics survey to produce an electronic form. Patients completed two UEQs (one for each type of audiometry) after finishing both audiometry sessions.'

In the same paragraph, the justification for using HHI, THI, and DHI in a diagnostic accuracy research may be included.

Response

The following has been added to the methods section to justify the use of these questionnaires:

'Objective measures do not always correlate with subjective symptoms and disability experienced varies among individuals with the same disease.(33) Use of questionnaires to assess disease burden can help address symptoms that are not identified by standard hearing tests. We wanted to observe if the presence of tinnitus affects TA performance, and how TA compares to hearing-related questionnaires for HL screening. Validated hearing-related questionnaires were completed by patients in between SBA and TA sessions: Hearing Handicap Inventory for Adults (HHIA, 25-item assessment of hearing impairment on emotional, social adjustments in adults), and if experiencing symptoms of tinnitus or dizziness, Tinnitus Handicap Inventory (THI, 25-item reviewing functional, emotional and catastrophic disability scales) and Dizziness Handicap Inventory (DHI, 25-item evaluating functional, emotional and physical domains).'

As observation has now been added to the results section:

'Based on BSA criteria in patients without apparent HL, 39% (12/31), 39% (12/31) and 58% (18/31) had HL, tinnitus and dizziness symptoms respectively, causing at least mild handicap or above based on questionnaire scores of HHIA>16, THI>16 and DHI>0. A similar trend was observed with ASHA criteria at 41% (17/41), 39% (16/41) and 51% (21/41) respectively in patients with no HL.'

What was the duration required to complete data collection for one participant, considering that the procedure included two tests and the administration of questionnaires?
Response

The following has been added:

'As SBA was part of the patient's standard of care, 30-45 minutes was allowed for TA, ototoxicity questionnaires and UEQs.'

Results

I believe that the analysis about ethnicity is unnecessary and recommend that the authors skip that section.

Response

The funder (NIHR) and the HRA (ethical approval committees) promote equality, diversity and inclusion in research (see links below) and as such our results show that recruitment was ethnically diverse, and the use of TA was acceptable across the cohort. We have therefore left this in.

Research inclusion | NIHR

Increasing the diversity of people taking part in research - Health Research Authority

In Table 3, Mean pure tone thresholds are analyzed. Nonetheless, I observed that these mean values pertain to the entire number of participants for each frequency. Is it feasible to do a sub-analysis by exclusively comparing individuals with high thresholds (hearing loss) at each frequency (>25 dB)? It will provide insight into the suitability of the TA relative to the SBA in identifying elevated thresholds.

Response

A sub-analysis of paired thresholds where SBA is according to BSA (>20dB) and ASHA (>25dB) criteria for hearing loss has been carried out. The tabulated results have been added to the supplementary material as Table S…. and the following to the results section:

'Sub-analysis of mean pure tone paired thresholds (Tables S5 and S6) comparing only where SBA thresholds were abnormal according to BSA (>20dB) and ASHA (>25dB) criteria for hearing loss revealed that six (0.5KHz, 1KHz, 2KHz, 6KHz, 8KHz, 10KHz) and five (0.5KHz, 2KHz, 6KHz, 8KHz, 10KHz) thresholds respectively remain significantly different compared to seven thresholds (0.25-4KHz, 8KHz, 12.5KHz) of all paired thresholds (Table 3).'

Supplementary material

Table S5 Mean pure tone thresholds per frequency with high hearing loss thresholds based according to BSA criteria (paired SBA >20dB)

Frequency		TA	SBA	r	95% CI	P value
	N=	TV (±SD) dB	TV (±SD) dB			
0·25KHz	56	36.79±16.14	37.95±13.97	0.90	-0.7328 to 3.054	.2245
0·5KHz	62	36.13±15.59	39.19±14.94	0.91	1.385 to 4.744	**.0005**
1KHz	63	36.67±17.04	38.81±15.31	0.94	0.6305 to 3.655	**.0062**
2KHz	70	36.07±15.95	38.64±15.67	0.90	0.8642 to 4.279	**.0037**
4KHz	104	39.47±15.20	40.34±14.70	0.91	-0.3723 to 2.103	.1685
6KHz	111	41.17±17.81	43.33±16.06	0.87	0.5008 to 3.824	**.0112**
8KHz	94	50.27±20.06	46.65±16.57	0.88	-5.606 to -1.628	**.0005**
10KHz	39	43.59±20.52	47.95±16.81	0.91	1.572 to 7.146	**.003**
12·5KHz	41	48.66±15.21	48.9±14.38	0.89	-1.987 to 2.474	.8262
16KHz	4	28.75±4.787	32.5±6.455	-0.67	-12.65 to 20.15	.5195

TA = Tablet-based audiometry, SBA = sound booth audiometry, KHz=KiloHertz, TV=Threshold value, SD=standard deviation, dB=decibel, CI=confidence interval, r= Pearson Correlation coefficient, %=percentage

Table S6 Mean pure tone thresholds per frequency with high hearing loss thresholds based according to ASHA criteria (paired SBA >25dB)

Frequency		TA	SBA	R	95% CI	P value
	N=	TV (±SD) dB	TV (±SD) dB			
0·25KHz	39	42.82±15.42	43.59±13.23	0.87	-1.656 to 3.194	.5247
0·5KHz	46	40.54±15.46	44.13±14.35	0.90	1.591 to 5.583	**.0007**
1KHz	42	43.81±16.34	45.71±14.42	0.93	-0.04048 to 3.85	.0547
2KHz	45	43.22±15.42	46.22±14.85	0.86	0.5736 to 5.426	**.0166**
4KHz	84	42.86±14.92	43.99±14.07	0.89	-0.3408 to 2.603	.1302
6KHz	91	44.89±17.27	47.36±14.97	0.85	0.5754 to 4.370	**.0112**
8KHz	83	52.95±19.49	49.52±15.49	0.87	-5.535 to -1.332	**.0017**
10KHz	37	45.14±19.88	49.19±16.35	0.90	1.171 to 6.937	**.0072**
12·5KHz	35	52.86±11.96	53±11.19	0.80	-2.373 to 2.659	.9088
16KHz	3	26.67±2.887	35±5.00	0.00	-6.009 to 22.68	.1296

TA = Tablet-based audiometry, SBA = sound booth audiometry, KHz=KiloHertz, TV=Threshold value, SD=standard deviation, dB=decibel, CI=confidence interval, r= Pearson Correlation coefficient, %=percentage

Table 4 and the third paragraph on page 8, beginning with "TA showed…," demonstrate the sensitivity and specificity of TA in relation to SBA across various frequency thresholds. I recommend that the author include sensitivity and specificity values for the following.

1. Comparison of Standard Pure Tone Averages (500 Hz, 1 kHz, and 2 kHz) in TA and SBA.
2. Comparison of High Frequency Averages (8 kHz, 10 kHz, 12.5 kHz) in TA and SBA.

Response

The reported sensitivity and specificity is that of TA compared to SBA for detection of HL according to BSA and ASHA criteria. The following has been added to the results section:

'There is higher sensitivity using the TA approach at EHF (8-12.5KHz) ranging from 85-95% (BSA) or 78-100% (ASHA) compared to low frequencies (0.5-2KHz) of 77-81% (BSA) or 80-88% (ASHA). Conversely specificity is lower at EHF (8-12.5KHz) between 86-95% (BSA) or 88-97% (ASHA) compared to low frequencies (0.5-2KHz) of 97-98% (BSA) or vs 97-98% (ASHA).'

On page 8, in the results section, how did the authors compare the outcomes of the User Experience Questionnaire? It is explicitly noted in the procedure that TA was automated and conducted by the participants themselves, while SBA was performed by an audiologist. Consequently, the participants would have lacked familiarity with the SBA, rendering a comparison of the user experiences between TA and SBA unsuitable. Given that the users were entirely different. For TA, the participants themselves were the users, whereas for SBA, the users were audiologists.
I recommend that the authors eliminate the comparison section (Figures B and C) from Table S1 (B and C). Instead, include further details regarding the user experience questionnaire items and the scoring criteria for each item. Which item score contributes to the aggregate scale score, etc.

Response

The end-user who ultimately experiences the hearing test is the patient and the UEQ is a quick way to measure patient's immediate feedback based on the process and interactions for SBA (audiologist and button) and TA (minimal human interaction with button on tablet). It is not a direct comparison but as TA is patient user based it was important to assess usability. The UEQ results of 3 different types of hearing test delivery were reported similarly in Vijayasingam A, et al. Thorax 2020;**75**:632–639. doi:10.1136/thoraxjnl-2019-214177. They display experiences between the various types of hearing tests making it easier to assess usability. We have therefore left the UEQ data unchanged in Figure 1 but adapted the results to reflect the different processes to say:

'Different user processes are involved with TA (minimal human interaction with button on tablet) and SBA (audiologist and button), with the usability analysis showing that TA demonstrated good levels of attractiveness and novelty, excellent perspicuity and efficiency, and above average dependability and stimulation scales (Figure 1). SBA displayed excellent perspicuity, above average efficiency and dependability, below average attractiveness and stimulation with poor novelty scales. The scale means are reported in Table S14, with Cronbach's alpha coefficient showing acceptable reliability with all scales.'

Discussion

On discussion page 11, the paragraph commencing with "although the use of..." is lengthy and elaborates on the shortage in hearing aid usage and its awareness. The paragraph can emphasize how the efficacy of hearing aid usage may be enhanced in the near future with the implementation of TA-based routine hearing screening programs.
Response

This paragraph has been shortened with equal emphasis on improving hearing aid uptake as well as the shortage of hearing aid use:

‘Digital technology such as TA could potentially improve societal HL detection if implemented in future routine hearing screening programs increasing accessibility especially in resource limited areas. HL is exacerbated by the time (usually 10 years) individuals accept that they have a hearing problem with the stigma of wearing hearing aids often associated with aging leading to delay.(45) Additional interventions such as guidance and internet-based hearing healthcare training should accompany those recommended hearing assist devices to encourage help-seeking health behaviour.(46,47) However, considerable challenges still remain with a secondary increased demand for aural rehabilitation services and requirement for hearing aid use. Within LMICs the cost of hearing aids can limit uptake in individuals or services who lack the resources to purchase, fit, deliver, maintain and support hearing aid use, as well as costs of batteries and travel to EHF facilities.(48–50) Health-economic modelling has suggested that increasing EHC services to cover 50% of the global population by 2030 would cost US$ 75 billion but would avert >110 million DALYs over 10 years.(1)’

The discussion may also encompass a comparison of Shoebox TA with any other currently available tablet-based hearing screener for adults that addresses extended high frequencies, such as the study by Brungart et al. (2017). Using tablet-based technology to deliver time-efficient ototoxicity monitoring. International Journal of Audiology, 57(sup4), S78–S86.
https://doi.org/10.1080/14992027.2017.1370138
Ehlert K, Heinze B, Graham MA, Swanepoel D. Surveillance for ototoxicity in platinum-based chemotherapy using mobile health audiometry with extended high frequencies. The Journal of Laryngology & Otology, 2023; 137(1): 61-67. doi:10.1017/S0022215122001281

Response

Brungart et al 2017 provides a short summary of two mobile audiometers used for ototoxicity monitoring (Oto-ID and Creare Wireless Audiometer) with no sensitivity/specificity data for direct accuracy comparison with Shoebox. Ehlert et al 2023 uses HearTest on Samsung A3 smartphone rather than TA and is not compared with SBA therefore there is also no sensitivity and specificity data. We are not able to directly compare data from our study in terms of sensitivity, specificity and accuracy with these two references. However, we have included a mention of these devices and the references in the discussion:

‘Other mobile technologies with EHF monitoring have been used as part of ototoxicity monitoring programmes to detect chemotherapy-induced ototoxicity (OtoID, touch screen portable audiometer, HearTest using Android systems), and noise-induced HL Creare (wireless audiometer) in boothless environments such as hospital clinics and the military.’

Another unique aspect of the paper is the inclusion of a user experience survey. Nevertheless, the findings were not addressed in the discussion. I recommend that the authors explore the results of the user experience survey and how it demonstrates the potential of the ShoeBox TA in facilitating the implementation of automated tablet-based EHF hearing screening programs for monitoring noise exposure and ototoxicity.
Response

A sentence has been added to the discussion about the UEQ results and how it would facilitate screening programmes:

As demonstrated by the UEQ scores, patients found TA easier to use, more efficient, more interesting, more innovative and highlights the usability of TA to facilitate with EHF monitoring into these hearing screening programmes.
